# Redox Control of the Dormant Cancer Cell Life Cycle

**DOI:** 10.3390/cells10102707

**Published:** 2021-10-09

**Authors:** Bowen Li, Yichun Huang, Hui Ming, Edouard C. Nice, Rongrong Xuan, Canhua Huang

**Affiliations:** 1 The Affiliated Hospital of Medical School of Ningbo University, Ningbo 315020, China; libowenvictor@outlook.com; 2 State Key Laboratory of Biotherapy and Cancer Center, West China Hospital and West China School of Basic Medical Sciences and Forensic Medicine, Sichuan University and Collaborative Innovation Center for Biotherapy, Chengdu 610041, China; minghui@stu.scu.edu.cn; 3 Clinical Medical College, Hubei University of Science and Technology, Xianning 437000, China; 17683751207@163.com; 4 Department of Biochemistry and Molecular Biology, Monash University, Clayton, VIC 3800, Australia; ed.nice@monash.edu

**Keywords:** cancer dormancy, redox signaling, cancer therapy, ROS

## Abstract

Following efficient tumor therapy, some cancer cells may survive through a dormancy process, contributing to tumor recurrence and worse outcomes. Dormancy is considered a process where most cancer cells in a tumor cell population are quiescent with no, or only slow, proliferation. Recent advances indicate that redox mechanisms control the dormant cancer cell life cycle, including dormancy entrance, long-term dormancy, and metastatic relapse. This regulatory network is orchestrated mainly through redox modification on key regulators or global change of reactive oxygen species (ROS) levels in dormant cancer cells. Encouragingly, several strategies targeting redox signaling, including sleeping, awaking, or killing dormant cancer cells are currently under early clinical evaluation. However, the molecular mechanisms underlying redox control of the dormant cancer cell cycle are poorly understood and need further exploration. In this review, we discuss the underlying molecular basis of redox signaling in the cell life cycle of dormant cancer and the potential redox-based targeting strategies for eliminating dormant cancer cells.

## 1. Introduction

The past three decades have witnessed rapid developments in cancer therapy, including advances in surgery, chemotherapy, radiotherapy, targeted therapy, and immunotherapy [1,2,3,4]. However, tumor metastasis and relapse may happen years or even decades after surgical eradication due to cancer cell dormancy, a process in which cells remain viable but cease to display or only display slow proliferation [5,6]. For cancer cells, this process is also called quiescence; when most cells in a tumor go through quiescence, it is called tumor dormancy. Cancer treatment may eradicate most cancer cells at the primary site, but some cancer cells may enter a quiescent state to survive and stop proliferation, which is considered one of the reasons for minimal residual disease [7]. At the same time, cancer cell dissemination from the primary tumor may occur at an early stage to form a remote dormant population, contributing to the recurrence of refractory tumors [8]. The beginning of disseminated cancer cell dormancy is considered a niche-dependent process when disseminated cancer cells engage with supportive niches, including bone marrow and metastatic and perivascular niches, where disseminated cancer cells go through a reprogramming process to enter dormancy [9,10]. After a cellular reprogramming process, the cancer cells enter into long-term dormancy [11]. When dormant cancer cells gather sufficient nutrients or are exposed to specific stimuli (for example, hormones, oxidative stress), the dormant cancer cells may reactivate and cause tumor relapse [12,13]. During secondary treatment on the relapsed tumor, the disseminated cancer cells may re-enter dormancy, which contributes to poor survival and a refractory state in late-stage patients [14]. Intriguingly, dormant cancer cells show similar characteristics to drug-tolerant cells, persister cells, and cancer stem cells, indicating that the regulation of cancer dormancy may rely on mutation-independent mechanisms, including epigenetic regulation or adaptive stress response [15,16,17,18]. For decades, researchers have widely recognized that the principle of dormant cancer cell life cycles is related to both intracellular signaling and extracellular stimuli [19], indicating that the control of the dormant cancer cell life cycle may contain a stress-dependent mechanism.

**Figure 1:**
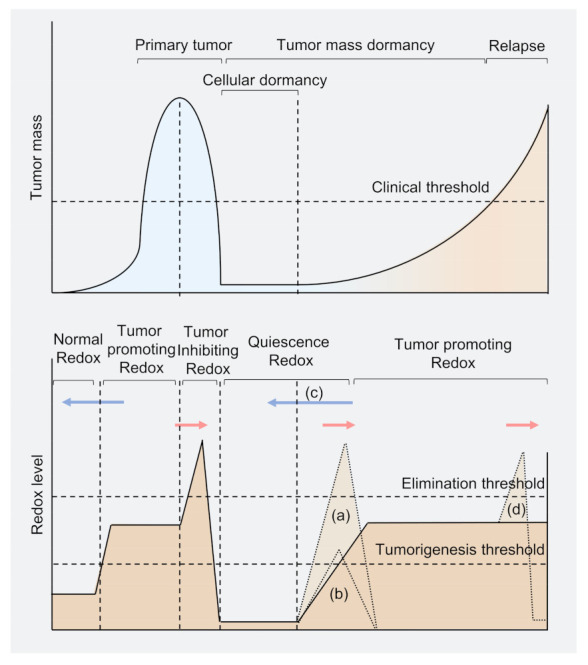
Model of tumor burden and redox level in cancer cell dormancy. Primary tumors rely on mild oxidative stress that increases ROS levels beyond the tumorigenesis threshold to proliferation. While under therapeutic conditions, the ROS level may be beyond the elimination threshold to rapidly decrease tumor mass. Several cancer cells (including residual cells and disseminated cells) may reprogram and survive in a redox level lower than normal cells and enter dormancy. When some dormant cancer cells awaken in the tumor population, the redox level may again exceed the tumorigenesis threshold. The tumor proliferates slowly under the clinical threshold, called tumor mass dormancy. Once the tumor mass exceeds the clinical threshold, the tumor has relapsed. Blue arrows represent potential strategies to conduct antioxidant therapy to treat tumors; red arrows represent potential oxidant-dependent therapy. (**a**) Strategy to kill dormant cancer cells through excessive ROS; (**b**) Strategy for reawaking dormant cancer cells to sensitize cancer cells to anti-proliferation drugs; (**c**) Strategy using antioxidant to keep dormant cancer cells from awaking; (**d**) Chemotherapy on recurrent tumors, but may enter into another life cycle of dormant cancer cells.

Oxidative stress is usually caused by the accumulation of reactive oxygen species (ROS). The intracellular sources of ROS are mainly generated from nicotinamide adenine dinucleotide phosphate (NADPH) oxidases, the mitochondrial electron transport chain (ETC), and endoplasmic reticulum (ER) stress. Extrinsic factors, including radiation, chemicals, or damage also play essential roles in ROS generation [20]. Increased ROS level may inactivate Kelch-like ECH-associated protein 1 (KEAP1), an essential component of E3 ubiquitin ligase complex targeting (NRF2), thus promoting the translation of antioxidant proteins, including superoxide dismutase (SOD), catalase, glutathione peroxidase (GPX), peroxiredoxins (PRX), thioredoxins (TRX), and glutathione reductase (GR), which maintains redox homeostasis [21,22]. Early studies of redox biology encouraged the notion that ROS are the toxic byproduct in cell metabolism, while recent advances provide provocative insights into their role as intracellular signaling molecules at a low physiological level [23,24]. Redox modifications on thiol of cysteine residues, including S-sulfenic acids (–SOH), S-sulfinic acids (–SO2H), and intramolecular or intermolecular disulfides (–SS–), can modulate protein structure, enzyme activity, and protein–protein interactions, which modulates key regulators in multiple signaling pathways [25,26]. ROS and antioxidants are in balance (redox homeostasis) in normal cells, while increased ROS levels are considered a hallmark of cancer cells, leading to genomic instability, facilitated cell proliferation, increased motility, and activated oncogenic signaling [24,27]. Excess ROS produced by chemotherapy may induce irreversible cell damage and cause tumor cell death [28,29]. However, some cancer cells may stay in quiescence by overexpressing antioxidant enzymes to maintain low levels of ROS, even lower than that of normal cells, to evade chemotherapy-induced cell death [30,31]. Such quiescent cancer cells are highly relevant to tumor recurrence and have several characteristics similar to dormant cancer cells, indicating potential relationships between redox and cancer dormancy [32]. Emerging evidence suggests that redox signaling is strongly associated with the dormant cancer cell life cycle (Figure 1). In this review, we focus on the mechanisms that control redox mechanisms in the dormant cancer cell life cycle, as well as strategies for targeting dormant cancer cells from a redox perspective.

## 2. Dormancy Entrance in Redox Perspective

**Figure 2:**
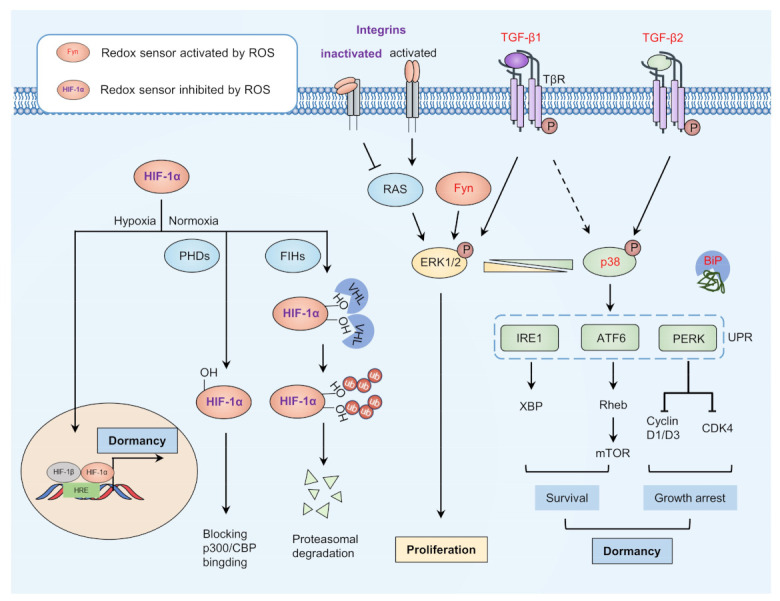
Model of dormancy entrance. The balance between proliferation and dormancy may be partly dependent on the ratio of p-p38 and p-ERK1/2. Redox can activate TGF-β2 and inactivate integrins to activate p38 and inhibit activation of ERK1/2, as well as to modify ER chaperone BiP for release from key regulators of ER-stress signaling, thus activating ER-stress signaling and promoting cell survival and growth arrest. Redox-activated TGF-β1 and Fyn can activate ERK1/2 to promote cell proliferation, while integrins activate when it is not redox-modified. HIF-1α is inhibited under normoxia through redox modification mediated by PHDs and FIHs. Under hypoxia conditions, HIF-1α may translocate to the nucleus and promote transcription of dormant-related genes. ER, endoplasmic reticulum; ERK1/2, extracellular signal-regulated kinase 1/2; FIHs, factor inhibiting HIF-1; HIF-1α, hypoxia-inducible transcription factors 1α; TGF-β2, transforming growth factor β2.

Disseminated cancer cells from the primary tumor site may transfer to a supportive niche for dormancy through intravascular processes [33,34]. Traditionally, researchers considered dissemination as a late stage of the metastatic cascade, which happened when cancer cells had acquired several key mutations to complete the colonization process [35]. However, multiple lines of evidence indicate that about 80% of metastasis is partly caused by cancer cell dissemination at an early stage, meaning that the dormant transform process may not be solely driven by genetic change [8,36,37]. Recent advances suggest that the regulation of dormancy entrance is closely related to redox signaling (Figure 2).

### 2.1. Balance between Dormancy and Proliferation

The ratio between the active form of two mitogen-activated protein kinases (MAPKs), extracellular signal-regulated kinase 1/2 (ERK1/2), and p38 are considered to be an indicator of cancer cell dormancy [38]. Specifically, the cancer cells favor proliferation when p-ERK1/2 play a dominant role (high p-ERK/p-p38 ratio); otherwise, the cancer cells prefer to enter dormancy (low p-ERK/p-p38 ratio). Recent studies indicate that a redox sensor tyrosine-protein kinase Fyn regulates both p-ERK1/2 and p-p38 through Ras/Raf/MEK/ERK pathway and MEK3/6, respectively. Intriguingly, the activation of p38 is considered redox-dependent rather than ERK1/2, since mutating the redox modified site Cys488 on Fyn turns the p38-relevant phenotype into ERK1/2-dominate phenotype [39]. Consistently, a study on global profiling of cysteine modification in *C. elegans* revealed that the enrichment score of p38 MAPK signaling was most significant [40], indicating that p38 MAPK signaling may take priority under redox regulation. Cys173 has been identified as a conserved redox-sensitive site for p38 activation regulation in *C. elegans* [40]. Furthermore, Cys119, and Cys162 in p38α also act as redox sensors, managing the formation of a heterodimer with mitogen-activated protein kinase kinase 3 (MKK3) [41]. However, the biological function and regulatory mechanisms of these redox-sensitive sites need further elucidation.

After activation of p38 under oxidative stress, the downstream ER-stress signaling may contribute to the quiescence of disseminated cancer cells [42,43]. Binding immunoglobulin protein (BiP) is an ER chaperone that binds under normal conditions with three downstream transducers of ER-stress signaling, including inositol-requiring enzyme 1α (IRE1α), activating transcription factor 6α (ATF6α) and proline-rich receptor-like protein kinase (PERK) [44]. When unfolded protein reaction (UPR) is induced, BiP then binds with misfolded peptides with release from ER transducers, thus activating ER-stress signaling [45]. Redox activated glutathione peroxidase 7 (GPx7) can oxidize BiP on Cys41 and Cys420 to form an intramolecular disulfide bond, resulting in an enhanced ability to bind to misfolded peptides and thus ER-stress signaling activation [46]. Redox-mediated activation of IRE1α and ATF6α may support dormant cancer cell survival, while activating PERK phosphorylates eukaryotic initiation factor 2α (eIF2α) and inhibiting its translation of cyclin-dependent kinases (CDK4), Cyclin D1/D3, leading to quiescence of dormant cancer cells [47]. These findings indicate that redox mechanisms may trigger dormant processes through redox modification on BiP, while redox modifications on other key regulators in ER-stress signaling need further exploration [48]. Moreover, other p38 downstream factors, including nuclear receptor subfamily 2 group F member 1 (NR2F1) and p53, may also participate in the entrance of cancer cell quiescence [49,50].

### 2.2. Reforming the Related Microenvironment

The tumor microenvironment contributes to tumor progression, immune evasion, metastasis, drug resistance, and dormancy, where redox reactions play a pivotal role [51,52]. Recent advances indicate redox regulates the entrance of cancer dormancy through regulating several key factors in the microenvironment, including hypoxia-inducible transcription factors (HIFs), integrins, and transforming growth factor β (TGF-β) signaling.

Due to the rapid growth of tumor cells and the aberrant formation of blood vessels, the tumor microenvironment exhibits hypoxia regions, resulting in cancer cell dormancy [53,54,55]. Redox controls hypoxic signaling by regulating the stability of HIFs. Under normal oxidative conditions, prolyl-4-hydroxylases (PHDs) hydroxylate HIF-1α on proline residues so that the von Hippel–Lindau protein (pVHL) polyubiquitinates HIF-1α and leads to 26S proteasome system-dependent degradation [56]. In addition, factor inhibiting HIF-1 (FIH-1) can hydroxylate HIF-1α on asparagine residues, thus preventing its binding to the p300/CBP complex and subsequent transcription [57]. While under hypoxia, HIF-1α can translocate into the nucleus and conduct transcription of dormant-related genes (for example, Cyclin G2). In addition, recent studies indicate constitutively photomorphogenic 9 signalosome subunit 8 (CSN8) can stabilize HIF-1α protein through deubiquitylation and upregulates HIF-1α transcription in a nuclear factor-κB (NF-κB)-dependent manner in the hypoxia tumor microenvironment, leading to epithelial–mesenchymal transition (EMT) and dormancy of colorectal cancer cells [58].

Apart from HIF-1α, redox in the tumor microenvironment can affect intracellular signaling by regulating several membrane receptors, including integrins and TGF-βreceptors, leading to cancer dormancy [59,60]. Redox can mediate intramolecular disulfide bond formation in integrins, promoting an inactivated bent conformation, resulting in cancer cell dormancy [61,62]. However, when the disulfide bond is reduced, integrins switch to the activated upright conformation, thus maintaining cell cycle progression through RAS-ERK/MAPK pathways or through cytoskeleton reorganization [63,64]. Multiple lines of evidence indicate that the TGF-β ligand secreted by tumor cells and niche cells in the tumor microenvironment can control cancer cell dormancy [59]. Of note, a recent advance shows that redox and TGF-β/ bone morphogenetic protein (BMP) signaling are sufficient to fully induce prostate cancer cell dormancy, indicating the pivotal role of redox in promoting cancer cell dormancy [65]. ROS can turn latent TGF-β into an active form and facilitate the phosphorylation of the TGF-β receptor I (ALK5), thus activate the TGF-β pathway [66,67]. Furthermore, ROS can inactivate phosphatase and tensin homolog (PTEN)/PPM1A-dephosphorylation of SMAD2/3, thus promoting the transcription of dormancy-inducing genes [68]. Intriguingly, activated TGF-β1 promotes T4-2 breast cancer cells to escape from dormancy in a 3D culture model while inhibiting T4-2 breast cancer cell proliferation in the 2D model, according to recent studies, which may be caused by the different redox states of these culture models [59,69]. Unlike TGF-β1, TGF-β2 to date has only been shown to exhibit its pro-dormant function in various cancer cells [70,71]. Other than intracellular signaling, redox effects can control TGF-β signaling in the local niches, thus affecting the dormancy of disseminated tumor cells. For example, bone marrow-derived TGF-β2 induces dormancy in both head and neck squamous cell carcinoma and a prostate cancer model in a tumor-specific and ligand-specific manner [70,71]. Moreover, redox controls the disulfide bond formation between homotrimers of thrombospondin-1 (TSP-1) in the perivascular niche, facilitating the activation of downstream TGF-β signaling and leading to quiescence of breast cancer cells [72]. Importantly, redox has the ability to disseminate cancer cells into dormancy by inactivating integrins or activating TGF-β2. Intriguingly, TGF-β1 exhibits a context-dependent function on cancer cell dormancy; how redox is involved in this process should be further evaluated.

## 3. Redox-Mediated Long-Term Dormancy

**Figure 3:**
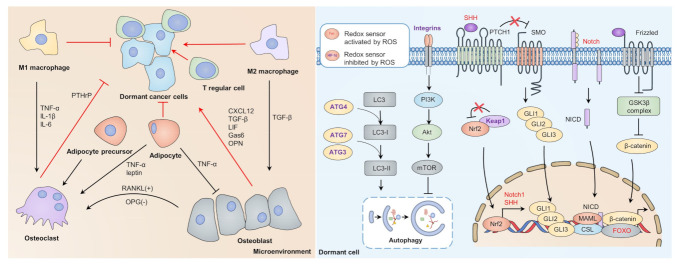
Dormant-dependent microenvironment and intrinsic supportive signaling. Interactions between immune cells and cytokines balance the entrance and escape of dormancy. Osteoblasts, M2 macrophages, and T regular cells are considered protectors of cancer dormancy, while osteoclasts, M1 macrophage, and adipocytes may break cancer dormancy. Redox control of dormant sustaining mechanisms. Redox may activate FOXO to bind with β-catenin to promote transcription of dormancy-related genes competitively. Upon oxidative stress, KEAP1 can be inactivated and release NRF2 to translocate into the nucleus, thus promoting transcription of Notch1 and SHH. Wnt, Notch, and Hedgehog signaling may sustain cancer dormancy through transcription of stemness-related genes. Oxidative stress may activate autophagy by impairing the integrin/PI3K/Akt/mTOR axis, which impairs autophagy by inactivating ATG4, ATG7, and ATG3. FOXO, forkhead box O; KEAP1, Kelch-like ECH-associated protein 1; mTOR, mammalian target of rapamycin; NRF2, nuclear factor erythroid 2-related factor 2; PI3K, phosphoinositide 3-kinase; SHH, sonic hedgehog.

When disseminated cancer cells enter the dormancy process, they have to keep themselves in quiescence and away from harmful stimuli. Redox mechanisms can sustain cancer cell dormancy in both an intrinsic (for example, sustaining stemness, inducing autophagy) and extrinsic way (immune cells in microenvironment) (Figure 3).

### 3.1. Redox Sustaining Cancer Cell Quiescence

Dormant cancer cells exhibit slow cycling, reversibility, and drug tolerance, thus sharing many similarities with cancer stem cells (CSCs) [17,73]. Recent advances indicate that redox-dependent intrinsic signaling (for example, Wnt signaling, Notch signaling, and Hedgehog signaling) plays an essential role in sustaining cancer stemness as well as cancer dormancy [74]. ROS mediates the nuclear translocation and affiliation between β-catenin and forkhead box O (FOXO) proteins by activating c-Jun N-terminal kinase (JNK) signaling, leading to apoptosis or quiescence [75,76]. When FOXO proteins are inactivated, β-catenin can promote proliferation by binding with T cell factor (TCF) transcription factors [77], suggesting the regulatory role of redox in Wnt signaling-dependent cancer dormancy. When cancer cells enter dormancy, the ROS levels may remain relatively low due to the high expression of antioxidant enzymes (for example, NRF2, SOD2) [32,78]. Intriguingly, NRF2 was found to coactivate with β-catenin in hepatocellular cancer (HCC), which may contribute to the refractory nature of HCC [79]. In addition, the expression of NRF2 may lead to downstream transcription of Notch1 and sonic hedgehog, which may activate Notch and Hedgehog signaling [80,81,82]. Multiple lines of evidence indicate the relationship between stemness-sustaining signaling and dormancy. For example, treating prostate cancer cells with Wnt5A promotes their dormancy in bone through the Wnt5A/ROR2/SIAH2 axis [83]. Itraconazole disrupts colorectal cancer dormancy by Wnt inhibition, indicating the essential function of Wnt signaling in dormancy maintenance [84]. In addition, IL-23 treatment induces cancer cell dormancy via Wnt/Notch signaling in esophageal squamous carcinoma cells (ESCCs) [85]. Moreover, Notch1 and Notch2 showed a regulatory role in breast cancer dormancy and recurrence [86,87]. The Hedgehog pathway also participates in cancer dormancy, inhibition of GLI2 abrogating leukemia stem cell (LSC) dormancy and chemoresistance [88]. These data suggest the pro-dormant role of stemness-sustaining signaling; however, the crosstalk and the detailed molecular mechanism involved in redox and quiescence still need further exploration.

Oxidative stress also modulates cancer cell dormancy through autophagy [89]. Autophagy is considered to be a lysosome-dependent degradation process to cope with stress [90]. Upon ER stress, autophagy can recycle misfolded proteins and damaged organelles, thus promoting survival signaling and maintaining dormancy [91,92]. In addition, redox can activate autophagy through the integrins/PI3K/Akt/mTOR axis and transcriptional control, thereby managing dormancy induction [63,93,94]. Intriguingly, oxidative stress also inhibits autophagy by multiple mechanisms. For example, the LC3 processer ATG4B showed attenuated activity after redox modification at Cys292 and Cys361, causing decreased autophagic flux [89,95]. Furthermore, oxidation of Atg3 and Atg7 abrogates phosphatidylethanolamine (PE) conjugation to microtubule-associated proteins 1A/1B light chain 3 (LC3), required for autophagy elongation in a mouse model [96]. Consistently, a hypoxic condition inhibits mTOR to activate autophagy [97]. The context-dependent role of ROS in regulating autophagy suggests that ROS may contribute to both entrance and abrogation of dormancy.

### 3.2. Redox Managing Dormant Microenvironment

Apart from intrinsic factors, the redox level in the microenvironment of the dormant cancer niche is also essential for the maintenance of cancer dormancy [98]. The microenvironment in dormant cancer niches is considered a complex system, where different immune cells and cytokines orchestrate the balance of the inflammatory or anti-inflammatory response [99]. For instance, osteoblasts may secrete CXC chemokine ligand 12 (CXCL12) [100,101], TGF-β [71], growth arrest-specific protein 6 (GAS6) [102], osteoprotegerin (OPG) [103], osteopontin (OPN) [104], and leukemia inhibitory factor (LIF) [105], to promote colonization and dormancy of disseminating cancer cells in bone marrow. Osteoclasts may play a dual role by secreting TGF-β to promote dormancy [106], while reverse dormancy occurs through parathyroid hormone-related protein (PTHrP) [107]. In addition, adipocyte precursors produce receptor activators of NF-κB ligand (RANKL) to stimulate osteoclast differentiation, thus awakening dormant cancer cells [108]. Similarly, adipocytes may promote osteoclastogenesis by secreting tumor necrosis factor α (TNF-α) [109] and may induce proliferation with leptin [110], which impairs the dormant state of quiescent cancer cells. Moreover, different subtypes of macrophages play different roles in modulating cancer dormancy through different secreted cytokines [111]. Furthermore, recent advances indicate that elevated CD73 in CD150^high^ CD4^+^ T cells and CD150^high^ T regulatory cells may protect dormant HSCs in the bone marrow niche from oxidative stress and immune elimination [112,113]. Managing redox level in the dormant microenvironment may become a potential strategy to control cancer dormancy. However, the crosstalk among these immune cells and inflammatory-dependent factors requires further study.

## 4. Redox Mechanisms Control the Metastatic Relapse of Dormant Cancer Cells

The entrance into and maintenance of cancer cell dormancy contribute to the survival of residual cancer cells after treatment, while the reactivation of dormant cancer cells is closely related to the metastatic relapse of various cancer types [6]. However, the mechanism of dormant cancer cell reactivation still needs further exploration. One possible explanation is that it is caused by both intrinsic and extrinsic factors. When disseminated cancer cells enter dormancy, they begin accumulating nutrients to support the reinitiation of tumor growth. Once they have gathered sufficient nutrients, the dormant cancer cells may trigger the reactivation program and maintain proliferation [19]. From the extrinsic aspect, adaptive stress responses, including hypoxia, oxidative stress, and UPR, have been shown to contribute to the reactivation of cancer cell dormancy [13].

### 4.1. Redox Regulates Metabolism-Related Reactivation of Dormant Cancer Cells

Although there are multiple studies on the proliferation/dormancy balance through the p-ERK/p-p38 ratio, direct studies on dormant cancer cell reactivation are still limited. Recent studies reveal that lipid metabolism participates in reactivation from dormancy [114]. For instance, the fatty acid translocase CD36 is upregulated in oral and prostate cancer cells, thus increasing the synthesis and uptake of β-oxidized lipid and driving metastatic outgrowth [115,116,117]. Intriguingly, redox modifications on Cys333 and Cys272 support the activity of CD36 and accelerate gastric cancer metastasis [118]. In addition, epoxyeicosatrienoic acids produced by P450 epoxygenases may be upregulated under oxidative stress, contributing to tumor dormancy escape [119]. Consistently, recent studies confirmed that oxidized lipids derived from stress-activated neutrophils reactivate dormant cancer cells during early recurrence in cancer [120]. Of note, NRF2 promotes reactivation of dormant breast cancer cells by sustaining redox homeostasis and nucleotide metabolism, indicating the importance of the antioxidant system in studying dormancy reactivation [32].

### 4.2. Redox Related Cell-Extrinsic Environmental Changes

Remodeling of the extrinsic environment is another pivotal cause of redox-mediated cancer dormancy escape [121,122]. Recent studies indicate that the induction of a microenvironment by nasal instillation of lipopolysaccharide (LPS) or tobacco smoke exposure causes the formation of neutrophil extracellular traps (NETs), leading to the proteolysis of the extracellular matrix (ECM) protein laminin and thrombospondin-1 (TSP-1) degradation, which mediates dormant cancer cells reactivation through integrin-dependent pathways [6,123]. For hormone-dependent breast cancer, estrogen can reactivate dormant breast cancer cells and promote cancer recurrence, where redox signaling may also play a pivotal role [124,125]. This mechanism may contribute to breast cancer relapse and the poor prognosis for recurrent breast cancer [12]. Moreover, remodeling of the endosteal niche by osteoclasts activates dormant myeloma cells [126]. Of note, sprouting neovasculature in the perivascular niche promotes dormancy escape through TGF-β1 and periostin derived from endothelial tip cells [72].

## 5. Targeting Dormant Cancer Cell Life Cycle by the Redox-Mediated Mechanism

**Figure 4:**
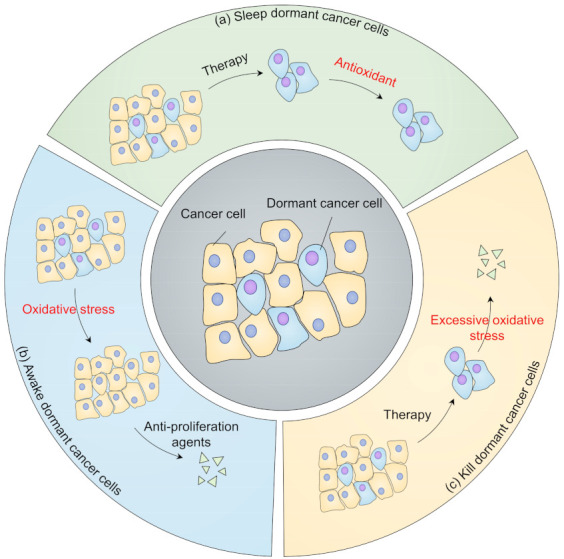
Strategies targeting dormancy from a redox perspective. (**a**) Keep dormant cancer cells asleep. After cancer therapy, dormant cancer cells may remain viable and remain at low redox levels. Antioxidant treatment may prevent dormant cancer cells from reactivating; (**b**) Awaken dormant cancer cells. Oxidants are used to reawaken dormant cancer cells, thus sensitizing them to anti-proliferation agents; (**c**) Kill dormant cancer cells. Oxidative phosphorylation inhibitors, autophagy inhibitors, and ferroptosis inducers can be used to eliminate dormant cancer cells.

Due to the limitation of any systematic studies on the redox state in dormant cancer cells, it is not yet possible to provide a precise strategy for targeting dormant cancer cells in different situations. However, there are typically three strategies to choose from for redox management, that is, targeting redox to sleep dormant cancer cells, awaking dormant cancer cells to increase the efficiency of anti-proliferation drugs, and eliminating dormant cancer cells through excessive oxidative stress (Figure 4).

### 5.1. Sleep or Reactivation in Dormant Cancer Cells

Preventing dormant cancer cells from reactivation by treatment with antioxidant agents may prolong the survival of patients who have a high probability of recurrence. Recent studies indicate that retinoic acid can reduce ROS under stress, sustaining hematopoietic stem cell (HSC) dormancy [127]. Of note, retinoic acid combined with 5-azacytidine as a dormancy-maintaining treatment has been evaluated in phase II clinical trial for head and neck squamous cell carcinoma (HNSCC) therapy (NCT03572387). For hormone-dependent prostate cancer and breast cancer, tumor recurrence is redox-dependent, contributing to poor patient survival [128,129]. A combination of the antioxidant muscadine grape extract with androgen deprivation therapy to treat recurrent prostate cancer has entered a phase II clinical trial (NCT03496805). Similarly, antioxidant green tea catechin extracts treating hormone receptor-negative stage I–III breast cancer are being evaluated in phase I clinical trial (NCT00516243). In addition, strategies to maintain cancer cell dormancy, including estrogen antagonists, inhibition of CDK4/6, ERK, and integrin signaling, may prevent reactivation of dormant cancer cells and tumor relapse, as shown by an acceptable clinical response in breast cancer [60,130,131,132]. However, targeting redox modification of key regulators in dormant signaling may lack specificity, which needs further exploration. Sustaining dormancy may require lifetime treatment, and some dormant cancer cells may not respond to therapeutic agents, causing low proliferation of tumors. 

Another strategy is to reactivate dormant cancer cells so that anti-proliferation agents can be used to inhibit these dormant cancer cells. For example, recent studies indicate that enhanced lipid metabolism sensitizes dormant cancer cells to 5-aminolevulinic acid-based photodynamic therapy [133]. Intriguingly, dormant HSCs undergoing acute interferon α (IFN-α) treatment are sensitive to anti-proliferation agents. Consistently, the combination of pegylated IFNs with nilotinib provides a better molecular response than nilotinib alone in chronic leukemia (CML) [134], and is under clinical evaluation for CML patients who have stable detectable molecular residual disease after imatinib treatment (NCT01866553). By contrast, chronic IFN-α treatment may cause HSCs to re-enter into the dormant state during chemotherapy, due to the different cellular ROS levels related to the two treatments [135,136,137]. Unlike strategies to keep dormant cancer cells asleep, reactivating dormant cancer cells may be more challenging, since activating dormant cancer cells is dose-dependent and could even promote tumor progression, limiting the clinical use of reactivation strategies. For example, a study on estrogen receptor alpha-positive dormant breast cancer treatment found that AMPK activated by metformin or high fatty acid intake may induce persistency of breast cancer cells under endocrine therapy; however, fatty acid oxidation inhibitors in combination with endocrine therapy may eliminate dormant cancer cells [138].

### 5.2. Killing Dormant Cancer Cells in a Redox Way

Recent advances indicate that dormant pancreatic ductal adenocarcinoma cells (PDACs) rely on oxidative phosphorylation, which is highly sensitive to the oxidative phosphorylation inhibitor oligomycin [139], for survival and recurrence. Similarly, researchers found that quiescent human leukemia stem cells (LSCs) are highly reliant on B-cell lymphoma 2 (BCL-2)-dependent oxidative phosphorylation; using BCL-2 inhibitors can selectively eradicate quiescent LSCs [140]. In addition, the novel oxidative phosphorylation inhibitor IACS010759 has been found to benefit patients with minimal residual acute myeloid leukemia (AML) cells after chemotherapy [141,142]. IACS010759 is entering phase I clinical trials for relapsed or refractory AML and other advanced cancers (NCT02882321, NCT03291938).

Impaired autophagy induced by hydroxychloroquine (HCQ), 3-methyladenine, or bafilomycin decreases the viability of dormant breast cancer cells through redox-dependent apoptosis, while for relapsed tumors, autophagy inhibition may increase the risk of metastasis, which indicates the context-dependent role of redox in these two cases [91,143]. A combination of autophagy inhibitor HCQ, CDK4/6 inhibitor Palbociclib, and the immune checkpoint programmed cell death-1 (PD-1)/Programmed death-ligand 1 (PD-L1) inhibitor avelumab to eradicate dormant breast cancer has entered a phase II clinical trial (NCT04841148). For patients who have detectable disseminated breast cancer cells in bone marrow, the combination therapy of HCQ and everolimus (NCT03400254) or the combination therapy of HCQ and gedatolisib (NCT03400254) are currently under clinical evaluation. Furthermore, the inhibition of ER-stress sensor PERK selectively eliminates dormant cancer cells under hypoxia conditions, suggesting targeting ER stress may also be a potential therapeutic strategy [144].

Ferroptosis is a redox-dependent process caused by redundant accumulated oxidized lipids, which has attracted much attention for tumor therapy [145,146]. Cancer cells that survive from targeted therapy or chemotherapy are efficiently eliminated by inhibiting glutathione peroxidase 4 (GPX4), the major antioxidant enzyme for reducing oxidated lipids [147,148]. Erlotinib, vemurafenib, and lapatinib eradicate PC9, A375, and BT474 cell lines through GPX4 inhibition, respectively, indicating the clinical potential of GPX4 inhibitors for dormant cancer therapy [148]. It is encouraging that GPX4 inhibitors [145], including altretamine and ashwagandha, are currently under clinical evaluation (NCT00002936, NCT04092647). In addition, aldehyde dehydrogenase (ALDH) can also sustain cancer survival under redundant lipid peroxidation by eliminating the downstream toxic aldehyde products [149]. The ALDH inhibitor crizotinib can abrogate the transition from toxic aldehydes to a harmless acid form, resulting in increased oxidative stress and the elimination of dormant cancer cells [150]. Other than GPX4 or ALDH inhibitors, there exist numerous strategies for inducing ferroptosis, including glutathione (GSH) inhibitors, iron activators, SLC7A11 inhibitors. The therapeutic effects of these strategies for killing dormant cancer cells need further evaluation.

## 6. Conclusions

In this review, we have summarized the recent advances in redox signaling in dormant cancer cells. Redox displays context-dependent roles in the control of the dormant cancer cell life cycle, indicating the complexity of the molecular mechanisms involved. Cancer cells rely on oxidative stress to promote proliferation and progression; however, under excess oxidative conditions (for instance, chemotherapy, radiotherapy), ROS may inactivate integrins and activate TGF-β2 on the membrane of some tumor cells, leading to the activation of p38 and downstream signaling (for example, ER-stress signaling), which ultimately causes cancer cell dormancy. Dormant cancer cells may highly express antioxidant enzymes, including NRF2, to maintain low ROS levels and upregulate stemness-sustaining signaling (for example, Wnt, Notch, Hedgehog signaling). During the dormant state, autophagy may cope with stress conditions to maintain cell quiescence. Apart from intracellular factors, the immune cells and cytokines in the dormant cancer microenvironment also participate in preserving cancer dormancy. When dormant cancer cells accumulate sufficient nutrients or suffer from oxidative stress from the extracellular microenvironment, cancer cells may escape from dormancy through activation of TGF-β1 or proliferation signaling. These highly reversible processes contribute to refractory or recurrent tumors, which currently lack efficient therapeutic strategies. From a redox perspective, there are three possible strategies: A combination of antioxidants (for example, retinoic acid, muscadine grape extract, green tea catechin extract) with targeted therapy to prevent reactivation of dormant cancer cells; Killing dormant cancer cells through excessive oxidative stress caused by inhibition of pro-survival signaling (oxidative phosphorylation and autophagy) and promoting ferroptosis; Combination of dormancy reactivating agents (for example, IFN-α) with an anti-proliferation agent. However, the latter has been limited in clinical use due to the difficulty of managing context-dependent redox levels and the risk of cancer progression. Encouragingly, several strategies have been shown to be effective and have entered clinical trials, which may favor the clinical treatment of cancer dormancy (Table 1).

Despite the rapid development of novel strategies to overcome dormant tumors, our molecular understanding of redox-dependent dormancy is still in its infancy. The main obstacle in studying redox-dependent dormancy is determining the redox threshold indicators in the dormant cancer cell life cycle (for example, dormancy entrance, maintaining, reactivation, and redox-dependent death). In addition, current strategies targeting redox are rather broad-based regulating methods, while targeted therapy and precision medicine are the current trends in cancer therapy. Exploring the biological function of redox modification on key regulators and the crosstalk between redox and dormancy-related signaling may assist the development of strategies for targeting allosteric disulfides to eliminate dormant tumors. Furthermore, since redox control of dormancy is context-dependent, intensive studies on redox-regulated networks in different organs and individuals should be considered. With increased understanding of the molecular mechanisms involved in clinical treatment, targeting dormant cancer cells through a redox perspective may shed new light on cancer therapy.

## Figures and Tables

**Table 1:** Summary of clinical trials targeting dormant cancer cells from redox perspectives.

Strategy	Title Name	Drug Name	Tumor	Phase	NCT Number
Keeping dormant cells asleep	Defined green tea catechin extract for treating women with hormone receptor-negative stage I-III breast cancer	Green tea catechin extract	Hormone receptor negative stage I–III breast cancer	I	NCT00516243
	Effects of muscadine grape extract in men with prostate cancer on androgen deprivation therapy	Muscadine Grape Extract, androgen deprivation therapy	Recurrent prostate cancer	II	NCT03496805
	A pilot study of 5-AZA and ATRA for prostate cancer with PSA-only recurrence after local treatment	5-Azacitidine, retinoic acid, Lupron	Prostate cancer	II	NCT03572387
Awaking dormant cells	Nilotinib Plus Pegylated Interferon-α2b in CML	Pegylated interferon α-2b, nilotinib	Chronic myeloid leukemia	II	NCT01866553
Killing dormant cells	IACS-010759 in advanced cancers	Oxidative Phosphorylation Inhibitor IACS-010759	Advanced cancers	I	NCT03291938
	Oxidative Phosphorylation Inhibitor IACS-010759 for treating patients with relapsed or refractory Acute Myeloid Leukemia	Oxidative Phosphorylation Inhibitor IACS-010759	Relapsed or refractory acute myeloid leukemia	I	NCT02882321
	Gedatolisib, Hydroxychloroquine, or the combination for prevention of recurrent breast cancer (“GLACIER”)	Hydroxychloroqui-ne, Gedatolisib	Breast cancer	I/II	NCT03400254
	CLEVER Pilot Trial: A phase II pilot trial of HydroxyChLoroquine, EVErolimus, or the combination for prevention of recurrent breast cancer	Hydroxychloroqui-ne, Everolimus	Breast cancer and harbored bone marrow disseminated tumor cells.	II	NCT03032406
	Avelumab or Hydroxychloroquine with or without Palbociclib to eliminate dormant breast cancer (PALAVY)	Hydroxychloroqui-ne, Avelumab, Palbociclib	Dormant breast cancer	II	NCT04841148
	Altretamine and Etoposide for treating patients with HIV-related cancer	Altretamine (GPX4 inhibitor), etoposide	HIV-related cancer	I	NCT00002936
	Ashwagandha for cognitive dysfunction	Ashwagandha (GPX4 inhibitor)	Breast cancer	II	NCT04092647

## Data Availability

No new data were created or analyzed in this study. Data sharing is not applicable to this article.
